# Diagnosis and severity criteria for sinusoidal obstruction syndrome/veno-occlusive disease in pediatric patients: a new classification from the European society for blood and marrow transplantation

**DOI:** 10.1038/bmt.2017.161

**Published:** 2017-07-31

**Authors:** S Corbacioglu, E Carreras, M Ansari, A Balduzzi, S Cesaro, J-H Dalle, F Dignan, B Gibson, T Guengoer, B Gruhn, A Lankester, F Locatelli, A Pagliuca, C Peters, P G Richardson, A S Schulz, P Sedlacek, J Stein, K-W Sykora, J Toporski, E Trigoso, K Vetteranta, J Wachowiak, E Wallhult, R Wynn, I Yaniv, A Yesilipek, M Mohty, P Bader

**Affiliations:** 1 Department of Pediatric Hematology, Oncology and Stem Cell Transplantation, University of Regensburg, Regensburg, Germany; 2 Hematology Department, Josep Carreras Foundation & Leukemia Research Institute, Hospital Clínic, Barcelona, Spain; 3 Hemato-Oncology Unit, Department of Pediatrics, University Hospital of Geneva, Geneva, Switzerland; 4 Pediatric Clinic, University of Milano-Bicocca, San Gerardo Hospital, Milan, Italy; 5 Department of Pediatric Oncohematology, Giambattista Rossi University Hospital, Verona, Italy; 6 Department of Hematology and Immunology, Hospital Robert Debre, Paris 7-Paris Diderot University, Paris, France; 7 Department of Clinical Haematology, Manchester Royal Infirmary, Manchester, UK; 8 Royal Hospital for Sick Children, Glasgow, UK; 9 Division of Blood and Marrow Transplantation, University Children’s Hospital, Zurich, Switzerland; 10 Department of Pediatrics, University Hospital of Jena, Jena, Germany; 11 Department of Pediatrics, Leiden University Medical Center, Leiden, The Netherlands; 12 Department of Pediatric Hematology and Oncology, University of Pavia, IRCCS Ospedale Pediatrico Bambino Gesù, Rome, Italy; 13 Department of Haematology, King’s College Hospital, London, UK; 14 Department of Pediatrics, St Anna Kinderspital, Vienna, Austria; 15 Division of Hematologic Malignancy, Department of Medical Oncology, Dana-Farber Cancer Institute, Boston, MA, USA; 16 Department of Pediatrics, University Children’s Hospital, Ulm, Germany; 17 Department of Pediatrics, University Hospital Motol, Prague, Czech Republic; 18 Schneider Children's Medical Center of Israel and Sackler Faculty of Medicine, University of Tel Aviv, Tel Aviv, Israel; 19 Pediatric Hematology-Oncology, Children’s Hospital, Medical School, Hannover, Germany; 20 Skåne University Hospital, Lund, Sweden; 21 University Hospital and Polytechnic La Fe, Valencia, Spain; 22 Children’s Hospital, University of Helsinki, Helsinki, Finland; 23 Department of Pediatric Hematology, Oncology and Hematopoietic Stem Cell Transplantation, University of Medical Sciences, Poznan, Poland; 24 Section of Hematology and Coagulation, Sahlgrenska University Hospital, Gothenburg, Sweden; 25 Royal Manchester Children’s Hospital, Manchester, UK; 26 Pediatric Stem Cell Transplantation Unit, Bahçeşehir University School of Medicine, Istanbul, Turkey; 27 Hôpital Saint-Antoine, APHP, Université Pierre & Marie Curie, INSERM UMRS 938, Paris, France; 28 Division for Stem Cell Transplantation and Immunology, Department for Children and Adolescents, University Hospital, Goethe University, Frankfurt/Main, Germany

## Abstract

The advances in hematopoietic cell transplantation (HCT) over the last decade have led to a transplant-related mortality below 15%. Hepatic sinusoidal obstruction syndrome/veno-occlusive disease (SOS/VOD) is a life-threatening complication of HCT that belongs to a group of diseases increasingly identified as transplant-related, systemic endothelial diseases. In most cases, SOS/VOD resolves within weeks; however, severe SOS/VOD results in multi-organ dysfunction/failure with a mortality rate >80%. A timely diagnosis of SOS/VOD is of critical importance, given the availability of therapeutic options with favorable tolerability. Current diagnostic criteria are used for adults and children. However, over the last decade it has become clear that SOS/VOD is significantly different between the age groups in terms of incidence, genetic predisposition, clinical presentation, prevention, treatment and outcome. Improved understanding of SOS/VOD and the availability of effective treatment questions the use of the Baltimore and Seattle criteria for diagnosing SOS/VOD in children. The aim of this position paper is to propose new diagnostic and severity criteria for SOS/VOD in children on behalf of the European Society for Blood and Marrow Transplantation.

## Introduction

Major advances in hematopoietic cell transplantation (HCT) over the last decade have substantially decreased transplant-related morbidity and mortality; the expected mortality rate is now less than 15%.^1^ Hepatic veno-occlusive disease (VOD), also called sinusoidal obstruction syndrome (SOS; referred to as SOS/VOD hereafter) belongs to a group of conditions increasingly designated as transplant-related, systemic endothelial diseases, that include acute GvHD, engraftment syndrome and transplant-associated microangiopathy (TAM). SOS/VOD is an unpredictable and potentially life-threatening complication of HCT.^2, 3^ The primary insult in SOS/VOD is injury to both sinusoidal endothelial cells and hepatocytes in zone 3 of the hepatic acinus,^4^ triggered by several factors, such as the toxicity of the conditioning regimen, release of cytokines due to inflammation and engraftment, release of endotoxins, phenomena of alloreactivity, protein C anticoagulant pathway abnormalities and use of calcineurin inhibitors. Furthermore, monoclonal antibodies tagged with calicheamicin derivatives, such as gemtuzumab ozogamicin and inotuzumab ozogamicin, are triggers of SOS/VOD and onset can occur after Ab administration alone or in subsequent HCT.^5, 6, 7^ Particularly in children, SOS/VOD can also occur as a complication of conventional radio- and chemotherapy outside of the transplant setting.^8, 9, 10, 11^ In addition to the triggers listed above, the risk of SOS/VOD is also dependent on patient-specific factors including genetic predisposition.^12, 13, 14^

While SOS/VOD usually resolves within weeks in most patients, an estimated 30–60% of affected children may progress to multi-organ dysfunction/failure (MOD/MOF).^4, 15, 16, 17^ In 20% of cases, SOS/VOD develops more than 30 days after HCT.^15, 18, 19^ The clinical presentation of SOS/VOD consists of hepatomegaly, ascites and weight gain. SOS/VOD is essentially a clinical diagnosis in the absence of sensitive and specific biologic markers or imaging tools.^20, 21, 22, 23, 24, 25, 26, 27^ Two sets of diagnostic criteria for SOS/VOD have been used in clinical practice for the past three decades:^15, 17^ the Baltimore^28^ and Seattle criteria,^29^ the latter subsequently modified by a number of minor adjustments.^15, 30, 31^ Based on these criteria, the incidence of SOS/VOD ranges between 10 and 60% in allogeneic HCTs with myeloablative conditioning (MAC) regimens, and between 5 and 30% in autologous HCT.^16^ SOS/VOD is seen significantly less often in patients who undergo reduced-intensity/toxicity conditioning regimens.^16, 32, 33, 34, 35^ In children, the average incidence of SOS/VOD is 20%, but in specific conditions can rise to 60%. This incidence is higher than that reported in adults.^15, 36, 37, 38^

Of note, the incidence of SOS/VOD differs by the criteria used for diagnosis, with up to a four-fold increased incidence of SOS/VOD observed between the Baltimore and Seattle criteria, respectively.^15, 16, 34^

The specific risk factors in children and the availability of effective licensed agents with favorable adverse-event profiles support the need for diagnostic and severity criteria specific to children.

### Rationale for new diagnostic criteria: are children different from adults?

Currently the same diagnostic criteria for SOS/VOD are used in adults and children. This is despite evidence that the disorder differs significantly between children and adults in terms of incidence, genetic predisposition, clinical presentation and the outcomes of prevention and treatment (Table 1). Such differences suggest that the currently used criteria are no longer appropriate for the diagnosis of SOS/VOD in children. The aim of this position paper is to propose diagnostic and severity criteria for SOS/VOD in pediatric patients on behalf of the European Society for Blood and Marrow Transplantation (EBMT).

In terms of treatment, several studies over the last two decades have demonstrated that defibrotide (DF) is effective for the treatment and prevention of SOS/VOD when used in combination with supportive care.^15, 38, 39, 40, 41, 42, 43, 44, 45^ DF has European Medicines Agency authorization for the treatment of severe SOS/VOD and most recently received approval from the Food and Drug Administration in the USA.^17, 46^ For treatment an international compassionate use program reported a superior outcome in children compared with adults.^47^ Early intervention with DF associated with a superior outcome was also shown first in children.^48^ The results of an expanded access Treatment-Investigational New Drug program, evaluating DF for the treatment of SOS/VOD, confirmed that earlier initiation of DF treatment was associated with significantly higher day +100 probability of survival.^49^

### Risk factors and incidence in children

Risk factors can be divided into those directly related to the transplant, those related to patient characteristics including the underlying disease, and those related to hepatic factors.^50^ Factors directly related to the pre-transplant regimen and transplant include the use of ozogamicin-linked antibodies (gemtuzumab and inotuzumab) prior to transplantation, use of drugs for conditioning known to be toxic to the endothelium (such as high-dose busulfan (BU), single-fraction or high-dose fractionated (⩾12 Gray) TBI-based conventional MAC,^16, 33, 35, 51, 52^ and/or a combination of BU and cyclophosphamide and/or melphalan) and prior HCT. All of the above predispose children to SOS/VOD.^15, 19^ Targeted IV BU has a predictable pharmacokinetic profile,^53, 54^ but the measurement of BU serum levels is complicated by differences in methodology and lack of a reliable target range for safety, in particular when given as part of combination chemotherapy. Therefore, targeted IV BU cannot guarantee a reduced incidence of SOS/VOD.^15, 55, 56, 57, 58^

In summary, although treatment-related risk factors are similar between children and adults, the influence of individual risk factors related to patient characteristics (such as age of onset of primary disease and potentially genotype^13^) is greater in children than adults.

A number of diseases, which are more common in childhood, can lead to an increased risk of SOS/VOD. For example, the reported incidence of SOS/VOD in infantile osteopetrosis is 60%.^15, 38, 59^ In congenital macrophage activation syndromes, such as familial hemophagocytic lymphohistiocytosis, Griscelli syndrome and X-linked lymphoproliferative disease, the reported incidence of SOS/VOD is 30%.^15, 60^ Patients with thalassemia with hepatomegaly and high iron overload have a reported incidence of 30–40%.^61, 62, 63^ High-risk neuroblastoma is currently treated in Europe with high-dose chemotherapy and autologous HCT, usually after conditioning with BU/melphalan. Compared with the <5% overall incidence of SOS/VOD following autologous HCT in adults,^16, 32^ the reported 15–30% incidence in children with neuroblastoma is exceedingly high.^15, 37, 58, 64, 65, 66^ SOS/VOD has been reported in children with Wilm's tumor, rhabdomyosarcoma and brain tumors given conventional chemotherapy, particularly in combination therapies including actinomycin D.^9, 67, 68, 69^ Sickle cell disease may also potentially carry a high risk of SOS/VOD during transplant due to a combination of a systemic vasculopathy, iron overload/liver fibrosis and hepatitis. Above all, with a reported overall incidence of 30%,^15, 70^ infants are probably the largest group at risk of SOS/VOD. This might be attributable to the immaturity of the liver in this age group.

In summary, the overall incidence of SOS/VOD in children and infants is between 22% and 30%, respectively, approximately two- to threefold higher than in adults. Under certain circumstances, the incidence can be as high as 60%, indicating that SOS/VOD is more prevalent in childhood.^15, 36, 37^

### Clinical presentation

The clinical presentation of SOS/VOD in children differs in several important respects from that in adults. The disease usually peaks around day +12 after HCT in both children and adults. However, while late-onset SOS/VOD is rare in adults,^19, 71, 72^ in children, 15–20% of SOS/VOD cases^15, 36, 37^ present beyond day +30, a time exceeding the 21- and 20- day limits specified by the Baltimore^28^ and Seattle criteria,^29^ respectively.

These sets of criteria^31^ also require a weight gain of 5 and 2%, respectively. In children a weight gain of 2% can be of doubtful significance. In particular, in infants, rapid weight changes due to iatrogenic fluid overload or inaccurate measurements, including the weight of clothing and diapers, can easily falsify results. Therefore, the Seattle criteria have been modified to require a 5% weight gain in children to improve specificity.^15^ However a single weight measurement does not reflect the dynamics of this disease.

The original publication of the Seattle criteria refers to ‘painful hepatomegaly’ as a criterion,^29^ whereas the subsequent publication lists ‘right upper quadrant pain’ as an independent criterion.^30^ In children, assessment of right upper quadrant pain is difficult, particularly in infants and toddlers, making this a subjective criterion in a high-risk pediatric patient population.

The *sine qua non* Baltimore criterion is hyperbilirubinemia ⩾2 mg/dL, which is an apparently objective and investigator-independent marker. However, anicteric SOS/VOD was observed in 32% of patients in the pediatric prevention trial, including those experiencing severe disease.^15^ This observation was confirmed in two independent publications, which reported an incidence of anicteric SOS/VOD of 30 and 29%, respectively.^73, 74^ Anicteric SOS/VOD seems to be particularly prevalent in children, although it is also seen in adults with late-onset SOS/VOD.^18, 19, 75^ Reflecting this, the new EBMT criteria for adults follow the Baltimore criteria of bilirubin ⩾2 mg/dL for early-onset SOS/VOD (<21 days), but omit hyperbilirubinemia as an obligatory criterion for late-onset SOS/VOD.^50^

Prompt diagnosis and early intervention are crucial in patients with SOS/VOD before they develop MOD/MOF with its associated high mortality rate.^34, 49^ Applying the Baltimore criteria for hyperbilirubinemia could mean that up to one-third of pediatric patients would currently fail to meet the diagnostic criteria of SOS/VOD and would therefore be excluded from timely and effective therapy. In terms of disease severity, hyperbilirubinemia is often a late finding in SOS/VOD, which reflects more advanced and perhaps irreversible hepatic injury, associated with a worse outcome. Naples *et al.*^74^ reported that the total treatment duration was longer and that the level of supportive care required was significantly higher in SOS/VOD patients with icterus compared with those without hyperbilirubinemia (*P*=0.224 and *P*=0.0081, respectively). In the pediatric prevention trial, the 60% incidence of MOD/MOF in patients who were diagnosed according to the Baltimore criteria was significantly higher than the 32% incidence seen in patients without hyperbilirubinemia (*P*=0.038).^15^ Carreras *et al.* reported on 845 allogeneic HCTs, where SOS/VOD was diagnosed using both the Seattle criteria, where bilirubin ⩾2 mg/dL is not diagnostic prerequisite, and the Baltimore criteria, with a cumulative incidence of 13.8% (*n*=117) and 8.8% (*n*=73), respectively. Of the 117 patients diagnosed according to the Seattle criteria, 37.6 (*n*=44) did not develop hyperbilirubinemia and therefore did not fulfill the Baltimore criteria; none of them developed MOD/MOF or had severe VOD. All MOD/MOF cases occurred in the group that fulfilled the Baltimore criteria.^34^ In a Japanese meta-analysis, the overall survival (OS) between patients diagnosed according to the Seattle or Baltimore criteria differed substantially.^35^ Jones *et al.*^28^ reported that only 1 out of 25 patients with VOD with a serum bilirubin level higher than 15 mg/dL survived. Finally many pediatric diseases, such as thalassemia, sickle cell disease, hemophagocytic lymphohistiocytosis, osteopetrosis and others, present with conjugated hyperbilirubinemia independently of HCT, so that a diagnostic limit of 2 mg/dL is inappropriate.

The specificity of the Baltimore and Seattle criteria have been reported to be 89 and 95%, respectively,^28, 29^ however, the sensitivity was low (56%).^76^ With an average 30% incidence of anicteric SOS/VOD in children, the estimate on specificity should be revised.

The recently revised diagnostic criteria for adults considered the inclusion of transfusion-refractory thrombocytopenia (RT) as a diagnostic criterion; however, this was excluded owing to the difficulty in evaluating RT during the pancytopenic phase after HCT.^50^ The current understanding of the pathophysiology of SOS/VOD is that a sustained and uncontrolled endothelial/sinusoidal activation is the primary triggering event. There is ample evidence that consumptive thrombocytopenia and refractoriness to platelet transfusions, most probably due to uncontrolled endothelial activation, is a hallmark of SOS/VOD.^28, 71, 77, 78, 79, 80, 81^ As early as 1993, McDonald *et al.*^30^ noticed an increased transfusion requirement before the onset of liver disease, most markedly in severe SOS/VOD. In clinical practice, after the exclusion of alternative causes, such as sepsis, TAM and other causes of consumptive thrombocytopenia, and Ab-mediated thrombocytopenia, the need for daily, or even more frequent platelet transfusion justifies a high level of suspicion of SOS/VOD.

### Imaging techniques

Hepatomegaly and ascites are two major diagnostic Seattle and Baltimore criteria, although the means of assessment have never been specified. Since publication of both sets of criteria there have been rapid advances in diagnostic imaging, such as computed tomography (CT), magnetic resonance imaging and ultrasonography (US). These objective tools can enable the accurate assessment of both liver size and the presence of ascites.

US is almost universally available, often as a bedside tool, and is therefore very useful for the confirmation of clinically suspected hepatomegaly and ascites. CT and magnetic resonance imaging can substitute for or complement US. Although both are less operator-dependent than US, their use is hampered by either exposure to radiation and/or the need for sedation in younger children. Furthermore, repetitive examinations to assess the dynamics of the disease are impractical and potentially harmful for immunocompromised children, as they would have to leave their protected environment, potentially exposing them to an increased risk of infection.

The diagnostic specificity of SOS/VOD has not been improved beyond hepatomegaly and ascites by the addition of hepatic artery resistive index, velocity of portal venous flow, increased periportal echogenicity and increased hepatic echotexture, as well as the assessment of gallbladder wall thickening.^27, 82, 83^

Measurement of the hepatic venous gradient pressure by cannulation of the jugular vein was demonstrated in adults to be a very accurate, but invasive, technique to confirm the diagnosis of SOS/VOD.^75, 84, 85^ Neither the efficacy nor safety of this procedure has been verified in children, therefore hepatic venous gradient pressure cannot be recommended as a tool for early diagnosis, particularly in infants and toddlers.

Doppler US assesses hepatic and portal vascular flow, pressure abnormalities and hepatic arterial early acceleration indices. Although they correlated best with HVPG, these techniques are subjective and very dependent on the experience of the operator. In addition, these findings, in particular portal venous flow reversal (hepatofugal flow), are either not consistently present or are a late finding, and might therefore be useful for the assessment of severity rather than early diagnosis.^26, 86, 87, 88, 89^ Most importantly, even in patients with severe and very severe SOS/VOD, hepatofugal flow can be absent, and should not be used as a criterion of exclusion.^90^

In summary, although serial clinical examination and weight monitoring remain the basic standard for assessing SOS/VOD, the most practicable imaging tool to confirm the clinical diagnosis of hepatomegaly and ascites is US. In general, baseline and serial measurements by any imaging tool are mandatory for early detection of any changes in liver size and/or presence of ascitic fluid. Similar to pre-existing hyperbilirubinemia, diseases, such as thalassemia, sickle cell disease, hemophagocytic lymphohistiocytosis or osteopetrosis and others, mostly transplanted during childhood, may present with pre-existing hepatomegaly and ascites before transplantation.

### New EBMT criteria for the diagnosis of SOS/VOD in children

The proposed EBMT criteria for the diagnosis of SOS/VOD in pediatric patients are summarized in Table 2. These criteria acknowledge the differences in various aspects of the disease between children and adults, and recognize the recent evidence of the superiority of early intervention. The proposals are based on reviews of the literature, and the experience and opinions of the authors. It is acknowledged that the proposed criteria should ideally be prospectively validated in clinical studies and tested in daily practice, with periodic reassessment to reflect changes in data and/or methodology.

As approximately one-fifth of the patients are diagnosed after 21 days post HCT, and reflecting the new EBMT criteria for adults,^50^ there should be no time limit for the diagnosis of late-onset SOS/VOD also in children.

Given the early onset of consumptive and transfusion-refractory thrombocytopenia in children, consumptive RT, defined as the need for at least daily platelet transfusions after the exclusion of other obvious causes, is viewed as an early and sensitive sign of SOS/VOD.

In children, daily measurements of weight and abdominal circumference may suggest the need for further investigations to assess hepatomegaly and ascites. A weight gain beyond 2% in a single measurement, as required by the Seattle criteria, can be misleading in children. Instead, the dynamics of endothelial damage and associated third spacing is better reflected by consecutive measurements over several days. Therefore, the pediatric EBMT criteria require a weight gain on 3 consecutive days, excluding other plausible causes, in addition to refractoriness to diuretic treatment. An unexplained weight gain of at least 5%, as required by the Baltimore criteria, will be retained.

Proper assessment of ascites and hepatomegaly and of their dynamics over time using radiation-free imaging techniques is recommended. This recommendation has the purpose of improving the sensitivity and specificity of the criteria, particularly in a patient population with a high prevalence of pre-existing ascites and hepatomegaly. For reasons of sensitivity and specificity, the decision should not be based on clinical examination only. A mandatory prerequisite is the comparability with a baseline examination. Therefore, the EBMT criteria suggest that the clinical diagnoses of ascites and hepatomegaly are verified with reproducible and objective diagnostic imaging tools. For the purpose of defining liver size and the amount of free abdominal fluid, US seems the most appropriate tool; CT and magnetic resonance imaging are acceptable but more complicated to perform in children.

As hyperbilirubinemia (>2 mg/dL bilirubin) in children is frequently either absent or found only in advanced-stage severe SOS/VOD^73, 74^ and because many children have pre-existing hyperbilirubinemia related to their primary disease, this criterion is difficult to use. For these reasons, pediatric EBMT criteria recognize anicteric SOS/VOD as a frequent entity and consider hyperbilirubinemia as a non-mandatory criterion. Instead of a predefined level of hyperbilirubinemia in children, the new EBMT criteria require the bilirubin level to rise from an individual baseline on 3 consecutive days, after the exclusion of competing causes.

Transjugular liver biopsy provides histologic evidence of SOS/VOD, and is frequently used in adults.^84, 85^ However, it is an invasive technique, difficult to perform in children and dangerous in patients with profound thrombocytopenia. In this respect, a recent study^91^ summarized a single institution experience on the safety and utility of liver biopsies after pediatric HCT. This study reported on 16 patients who received 18 liver biopsies. Five of these were by the transjugular route. Complications occurred after five biopsies, four of which were transjugular. The most common complication was hemorrhage. Two of these patients required transfer to the intensive care unit for procedure-related complications.^91^ This experience suggests that transjugular liver biopsies should be avoided in children.

### New EBMT criteria for grading the severity of SOS/VOD in children

The implementation of pediatric EBMT criteria for SOS/VOD offers the potential for early therapeutic intervention with effective and well-tolerated drugs, which may impact on the presentation, incidence of severity and outcome of SOS/VOD. Consequently, the implementation of severity criteria for children is mandatory. Chao *et al.*^92^ and Carreras *et al.*^75^ proposed a grading system for adults implementing the five categories from the CTCAE (Common Terminology Criteria for Adverse Events): grade 1=mild, grade 2=moderate, grade 3=severe, grade 4=very severe and grade 5=death, which was adopted in the new severity criteria for adults.^50^ The proposed criteria for severity grading of SOS/VOD in pediatric patients are summarized in Table 3.

Owing to the primarily endothelial/sinusoidal nature of the disease, elevated transaminases are not usually found in the early stages of SOS/VOD when primary hepatocytic toxicity, such as infections and drug toxicity, should be excluded. Rising transaminases after the diagnosis of SOS/VOD reflect hepatocyte failure and advanced-stage disease (CTCAE level 4). Glutamate dehydrogenase is a liver-specific enzyme with mitochondrial expression, particularly in the central, perivenular hepatocytes of the liver lobule (zone 3 of the hepatic acinus). As such, an elevation of glutamate dehydrogenase may reflect severe hepatocellular damage and could be considered a more reliable assessment of severity in SOS/VOD in the future.

Persistent RT for >7 days despite early therapeutic intervention represents severe disease.^30^

Bilirubin ⩾2 mg/dL is associated with morbidity and a worse OS despite pre-emptive therapeutic intervention,^15, 73, 74^ and therefore defines severe SOS/VOD in children.

Ascites and third spacing during the course of SOS/VOD have been recognized as criteria of severity. This mirrors the Bearman criteria, according to which early onset or marked weight gain were considered diagnostic of severe SOS/VOD.^79, 93^ A precise measurement of ascitic fluid is not possible, but the need for paracentesis to release abdominal pressure and alleviate respiratory distress is a criterion of severe SOS/VOD. Furthermore, repeated drainage of peritoneal fluid over a defined period is a sign of uncontrolled disease.

A combination of two of the following criteria: persistent ascitic drainage, persistent RT, sustained high levels of liver function indexes (transaminases, glutamate dehydrogenase) and bilirubin ⩾2 mg/dL defines very severe SOS/VOD (CTCAE grade 4) and predicts for an increased risk of death and the need for prolonged treatment.

Similar to the severity criteria for adults, the kinetics of symptom presentation is critical to evaluating the severity of SOS/VOD in children. The combination of several of the SOS/VOD-related signs mentioned above, within 48 h, is likely to lead to severe SOS/VOD.^75, 79, 93^ In particular, a doubling of the bilirubin level from an individual baseline within 48 h should be considered a sign of severe SOS/VOD.

Patients requiring replacement of coagulation factors after the established diagnosis of SOS/VOD are considered to suffer from consumptive coagulopathy associated with hepatic failure.

Severe SOS/VOD resulting in MOD/MOF, is characterized by pulmonary and/or renal dysfunction and/or encephalopathy.^16, 17^ Any combination of organ failure in SOS/VOD classifies very severe disease.

## Conclusions

By defining specific diagnostic and severity criteria for SOS/VOD in children, the group of EBMT experts aimed to overcome the weaknesses associated with the currently used criteria. This modification acknowledges both the pediatric-specific risk factors for SOS/VOD and the availability of an effective treatment option for children. Similar to the new EBMT criteria for adults, the group acknowledges that these proposals should be tested in daily practice and prospectively validated in clinical trials. These new criteria must stand the test of time and be periodically re-assessed, particularly biomarkers and imaging tools.

In summary, implementation of the proposed EBMT criteria for diagnosis and assessment of the severity of SOS/VOD will hopefully lead to earlier identification of patients in need of pre-emptive intervention with effective drugs for the treatment of SOS/VOD, and to the possibility of a predictive assessment of treatment effectiveness and outcome.^94^

## Figures and Tables

**Table 1:** Major differences in hepatic SOS/VOD between adults and children

*Criteria*	*Children*	*Adults*
Incidence	• Approximately 20% • Up to 60% in high-risk patients	• Approximately 10%
Risk factors	Additional pediatric risk factors: • Infants • Pediatric/genetic diseases with incidences above average	• Established risk factors
Clinical presentation	• Late-onset SOS/VOD in 20% • Anicteric SOS/VOD in 30% • Hyperbilirubinemia, if present: ○ Is frequently pre-existent ○ Occurs late during SOS/VOD ○ Is typical of severe SOS/VOD	• Late-onset SOS/VOD is rare • Anicteric SOS/VOD is rare
Need for proper assessment of ascites and hepatomegaly	• High incidence of disease-related hepatomegaly and ascites pre-HCT	
Treatment	• DF for severe SOS/VOD with MOD/MOF was associated with better results in children compared with adults	
Prevention	• DF demonstrated efficacy for prevention of SOS/VOD in children in a randomized prospective trial	

Abbreviations: DF=defibrotide; HCT=hematopoietic cell transplantation; MOD/MOF=multi-organ dysfunction/multi-organ failure; SOS/VOD=sinusoidal obstruction syndrome/veno-occlusive disease.

**Table 2:** EBMT diagnostic criteria for hepatic SOS/VOD in children

• No limitation for time of onset of SOS/VOD
The presence of two or more of the following^a^
• Unexplained consumptive and transfusion-refractory thrombocytopenia^b^
• Otherwise unexplained weight gain on three consecutive days despite the use of diuretics or a weight gain >5% above baseline value
• cHepatomegaly (best if confirmed by imaging) above baseline value
• cAscites (best if confirmed by imaging) above baseline value
• Rising bilirubin from a baseline value on 3 consecutive days or bilirubin ⩾2 mg/dL within 72 h

Abbreviations: CT=computed tomography; HCT=hematopoietic cell transplantation; MRI=magnetic resonance imaging; SOS/VOD=sinusoidal obstruction syndrome/veno-occlusive disease; US=ultrasonography.

a With the exclusion of other potential differential diagnoses.

b ⩾1 weight-adjusted platelet substitution/day to maintain institutional transfusion guidelines.

c Suggested: imaging (US, CT or MRI) immediately before HCT to determine baseline value for both hepatomegaly and ascites.

**Table 3:** EBMT criteria for grading the severity of suspected hepatic SOS/VOD in children^a^

*CTCAE*	*Mild*	*Moderate*	*Severe*	*Very severe MOD/MOF*
	*1*	*2*	*3*	*4*
LFT^b^ (ALT, AST, GLDH)	⩽2 × normal	>2 and ⩽5 × normal	>5	
Persistent RT^b^	<3 days	3–7 days	>7 days	
Bilirubin (mg/dL)^b^^,^ c	<2		⩾2	
Bilirubin (μmol/L)	<34		⩾34	
Ascites^b^	Minimal	Moderate	Necessity for paracentesis (external drainage)	
Bilirubin kinetics				Doubling within 48 h
Coagulation	Normal	Normal	Impaired coagulation	Impaired coagulation with need for replacement of coagulation factors
Renal function GFR (mL/min)	89–60	59–30	29–15	<15 (renal failure)
Pulmonary function (oxygen requirement)	<2 L/min	>2 L/min	Invasive pulmonary ventilation (including CPAP)	
CNS	Normal	Normal	Normal	New onset cognitive impairment

Abbreviations: ALT=alanine transaminase; AST=aspartate transaminase; CNS=central nervous system; CPAP=continuous positive airway pressure; CTCAE=Common Terminology Criteria for Adverse Events; GFR=glomerular filtration rate; GLDH=glutamate dehydrogenase; LFT=liver function test; MOD/MOF=multi-organ dysfunction/multi-organ failure; RT=refractory thrombocytopenia; SOS/VOD, sinusoidal obstruction syndrome/veno-occlusive disease.

a If patient fulfills criteria in different categories they must be classified in the most severe category. In addition, the kinetics of the evolution of cumulative symptoms within 48 h predicts severe disease.

b Presence of ⩾2 of these criteria qualifies for an upgrade to CTCAE level 4 (very severe SOS/VOD).

c Excluding pre-existent hyperbilirubinemia due to primary disease.
